# The influence of astrocytic leaflet motility on ionic signalling and homeostasis at active synapses

**DOI:** 10.1038/s41598-023-30189-8

**Published:** 2023-02-21

**Authors:** Marinus Toman, John Joseph Wade, Alexei Verkhratsky, Mark Dallas, Angela Bithell, Bronac Flanagan, Jim Harkin, Liam McDaid

**Affiliations:** 1 Computational Neuroscience and Neuromorphic Engineering Team, Intelligent Systems Research Centre, Ulster University, Northland Road, Derry, BT48 7JL UK; 2 Faculty of Biology, Medicine and Health, University of Manchester, Manchester, UK; 3 Achucarro Center for Neuroscience, IKERBASQUE, Basque Foundation for Science, Bilbao, Spain; 4 Department of Stem Cell Biology, State Research Institute Centre for Innovative Medicine, 01102 Vilnius, Lithuania; 5 Reading School of Pharmacy, University of Reading, Reading, UK

**Keywords:** Biophysical models, Dynamical systems, Astrocyte, Cellular neuroscience, Ion channels in the nervous system, Cellular signalling networks

## Abstract

Astrocytes display a highly complex, spongiform morphology, with their fine terminal processes (leaflets) exercising dynamic degrees of synaptic coverage, from touching and surrounding the synapse to being retracted from the synaptic region. In this paper, a computational model is used to reveal the effect of the astrocyte-synapse spatial relationship on ionic homeostasis. Specifically, our model predicts that varying degrees of astrocyte leaflet coverage influences concentrations of K^+^, Na^+^ and Ca^2+^, and results show that leaflet motility strongly influences Ca^2+^ uptake, as well as glutamate and K^+^ to a lesser extent. Furthermore, this paper highlights that an astrocytic leaflet that is in proximity to the synaptic cleft loses the ability to form a Ca^2+^ microdomain, whereas when the leaflet is remote from the synaptic cleft, a Ca^2+^ microdomain can form. This may have implications for Ca^2+^-dependent leaflet motility.

## Introduction

Astrocytes have a highly complex morphology with multiple primary processes (defined as branches) emanating from the soma and many peripheral processes known as leaflets, which originate from branches of various orders. Astrocyte leaflets are extremely thin, delicate and are beyond the resolution threshold of optical microscopy^1^. These leaflets have a heterogeneous structure, with some processes having a tube-like or sheet-like structure. The leaflets are < 100 nm in width with an exceptionally high (> 25 μm^−1^) surface-to-volume ratio^2^. They are rarely studied in live tissue as they are not directly accessible to electrophysiology, cannot be isolated for biochemistry, and are smaller than standard optical microscopy resolution. Perisynaptic astrocytic structures have been imaged on the sub-micron scale and were observed at varying levels of synaptic spatial association^3,4^, from touching a synapse and surrounding it almost completely, to being retracted from the synapse region and providing very little coverage. Transitions between these extremes accompany various physiological processes including synaptic plasticity^5^. Astrocytic coverage of synapses occurs in many regions of the brain including the cerebellum, cortex and hippocampus^6,7^.

Astroglial compartments that surround synapses are known as perisynaptic cradles (PsCs), which ensure local specificity of synaptic transmission by preventing synaptic cross-talk. PsCs are also responsible for the monitoring and maintenance of the synaptic microenvironment by regulating ionic and neurotransmitter concentrations in the synaptic cleft^8^. The small diameter of leaflets is likely to affect ionic distribution within their cytosol, which in turn may create functionally independent compartments^9^. Identifying such functionally independent compartmentalisation within parts of the central nervous system (CNS) is likely to be a significant and currently overlooked aspect towards a more complete understanding of the activity of neural networks.

Buffering of K^+^, carried out primarily by astrocytes, is essential for CNS ionostasis^10^. During the repolarisation phase of a neuronal action potential, K^+^ is released into the extracellular space and without K^+^ clearance, excess extracellular K^+^ ([K^+^]_o_) can affect neuronal excitability^11^. Astrocytes accumulate K^+^ via Na^+^-K^+^-ATPase (NKA); the astrocytic NKA assembly contains α2 subunits highly sensitive to physiological fluctuations of [K^+^]_o_. After cessation of neuronal activity, astrocytes shuttle K^+^ back by diffusion through K^+^ inward rectifying channels (K_ir_4.1) to restore neuronal ionic gradients. Astrocytes have a high density of NKA pumps and K_ir_4.1 channels clustered at the PsC^12^. To operate the astrocyte-neuronal K^+^ shuttle, K^+^ must be stored locally in microdomains within the PsC^13^. Ionic microdomains typically form within the cell close to the cell membrane and because astrocytic leaflets have a high surface-to-volume ratio and a poorly conducting leaf-like structure, K^+^ microdomains emerge in the PsC^12^.

The focus of this paper is to develop a computational model that captures astrocyte leaflet motility and its effect on ionic homeostasis. The model will show that varying degrees of synaptic coverage by the astrocyte leaflet has a significant effect on the concentration of different ionic species in both the astrocyte leaflet and the synaptic cleft. Furthermore, results show that an astrocytic leaflet that is in proximity to the synaptic cleft lacks the ability to retain a Ca^2+^ microdomain but K^+^ uptake is enhanced. Conversely, when the leaflet is remote from the synaptic cleft, a Ca^2+^ microdomain can form whereas K^+^ uptake becomes less effective and therefore the efficiency of the astrocyte K^+^ shuttle is also reduced.

This paper initially focuses on modelling ionic homeostasis where the extracellular space (ECS) volume is fixed. This allows a model to be developed that captures K^+^ clearance where it is assumed K^+^ ions released presynaptically are remote from the synapse active region and are therefore not considered: only K^+^ ions released from the postsynaptic neuron are considered in the proposed model. Also, the transport model proposed for the excitatory amino acid transporter (EAAT) is based on Michaelis–Menten kinetics and is a more accurate representation of physiological conditions. As the focus of the paper is to investigate ionostasis when ECS volume is changing, this model is used to simulate K^+^, Na^+^, Ca^2+^ and glutamate (Glu) dynamics for different ECS volumes.

## Methods

**Figure 1:**
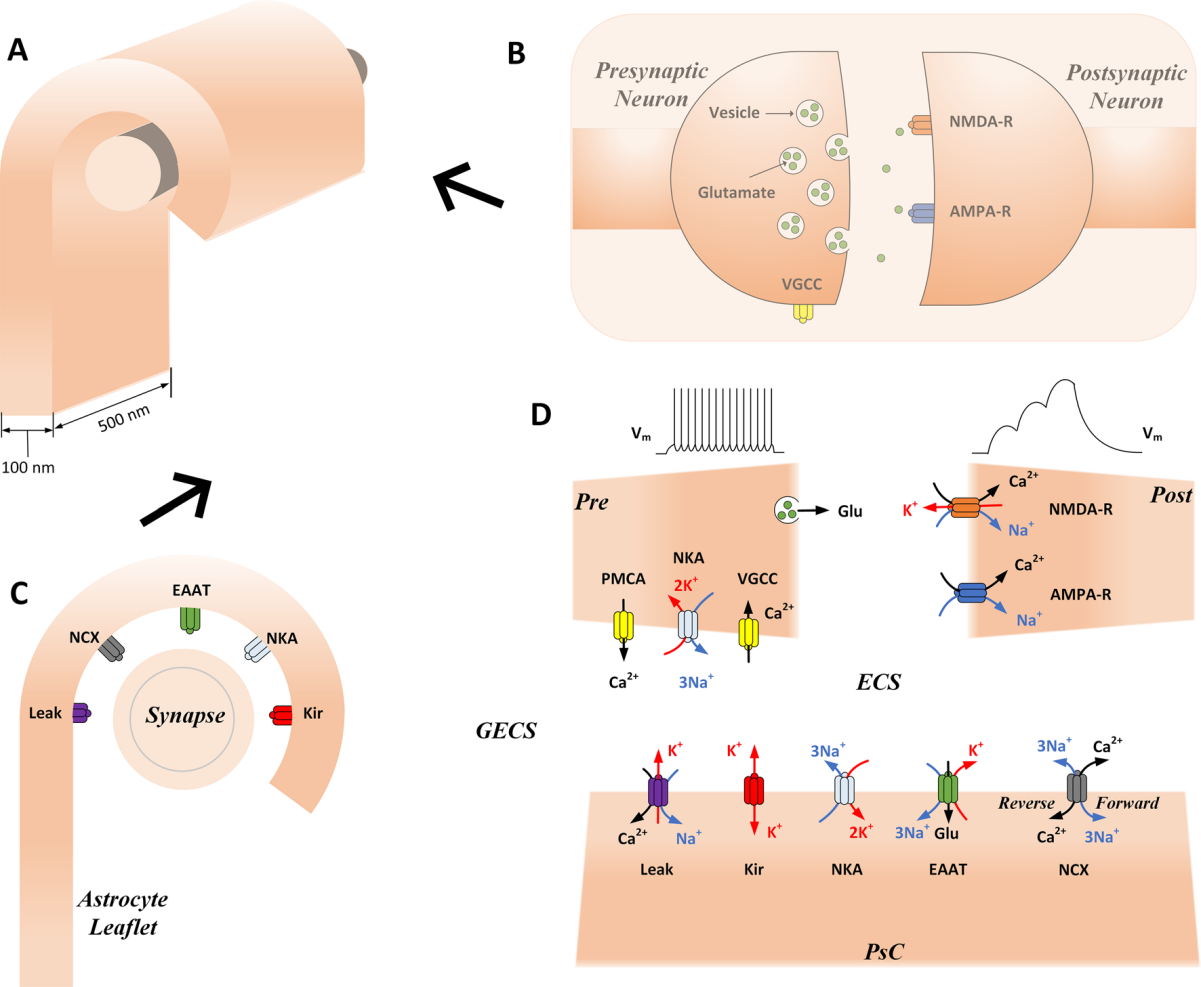
Schematic showing leaflet wrapping around synapse with the transmembrane channels, transporters and receptors used in the model. 3-D representation of an astrocyte leaflet enwrapping a neuronal synapse (**A**), with the right-side view (**B**) showing the main synaptic channels and receptors, and the front view (**C**) showing the astrocytic channels, transporters and exchangers. A schematic is given to show the signalling pathways and the compartments used in the model (**D**). Glu is released from the presynaptic terminal into the synaptic cleft and activates Glu receptors (AMPA-R and NMDA-R) on the postsynaptic dendritic spine. Glu also binds/unbinds to/from EAATs which actively transport Glu away from the cleft into the PsC. K^+^ is released into the cleft from the NMDA-R and through the astrocytic EAATs and K_ir_4.1 channels. K^+^ is taken up by the PsC through the NKA pump. K^+^ flows away from the perisynaptic cradle in an extremely restricted manner along the ultra-thin astrocyte leaflet. Na^+^ and Ca^2+^ channels are also shown; astrocytic NCX and neuronal VGCC and plasma-membrane Ca^2+^-ATPase (PMCA). The NCX extrudes Ca^2+^ under physiological conditions, however, the NCX can readily reverse to extrude Na^+^ and uptake Ca^2+^. During an action potential, the VGCC opens to allow an influx of Ca^2+^ which promotes Glu release. The PMCA acts much like the NKA, using the energy from ATP to maintain the Ca^2+^ gradient. For clarity, only active channels are shown in the schematic, however for model completeness, neuronal passive transmembrane channels and extracellular diffusive currents are also included in the model which are not shown in the schematic.

The proposed model is an extension of the previous one, which predicted the formation of local Ca^2+^ microdomains in PsCs due to the reversal of the Na^+^/Ca^2+^ exchanger (NCX) during neuronal activity^14^. The present model is a five-compartment conductance-based model consisting of (i) a presynaptic axon terminal (Pre), (ii) a postsynaptic dendritic spine (Post), (iii) a local extracellular space (ECS), (iv) a PsC and (v) a global extracellular space (GECS). Neuronal and astrocytic compartments have various ionic channels and transporters dwelling in their respective membranes, which are modelled using well-established conductance-based equations. The compartments and ionic channels used in the model are shown in Fig. 1. The compartments are assumed to have uniformly distributed concentrations of three ionic species: Na^+^, K^+^ and Ca^2+^. The model consists of six key variables which control the state of the synapse: the concentrations of Na^+^, K^+^ and Ca^2+^ in the PsC and ECS compartments. The GECS is assumed to have a much larger volume than the ECS and therefore the concentrations of all ions are held constant in the GECS. Concentration changes are described in terms of transmembrane currents produced by the electrogenic ion channels interfacing with each compartment. Concentration changes take the general form:

1 $$\begin{array}{c}\frac{d{\left[x\right]}_{y}}{dt}=-\left(\frac{{I}_{x}}{{z}_{x}F Vo{l}_{y}}\right)\end{array}$$

where *x* is the ion under consideration (Na^+^, K^+^ and Ca^2+^), *y* is the compartment under consideration (Pre, Post, PsC and ECS), *I*_*x*_ is the total current of ion *x* entering/leaving the compartment *y*, *z*_*x*_ is the ionic valence, *F* is Faraday’s constant and *Vol*_*y*_ is the volume of compartment *y*. The equations and parameters for the currents of the channels shown in Fig. 1 are given in Supplementary Tables S1 and S2. The membrane potential of each neuronal cell is described using a standard Hodgkin-Huxley type ohmic equation:

2 $$\begin{array}{c}{C}_{m}\frac{d{V}_{y}}{dt}={I}_{ext}-I\end{array}$$

where *C*_*m*_ is the membrane capacitance, *V*_*y*_ is the membrane potential of compartment *y*, *I*_*ext*_ is an externally applied current and *I* is the total transmembrane current. Our previous model used a single-compartment synapse^14^, whereas here, we split the synapse into two separate compartments: Pre and Post. The Pre compartment has voltage-gated Ca^2+^ channels (VGCCs) necessary for exocytosis of Glu and the Post compartment has Glu receptors necessary for successful synaptic transmission: N-methyl-d-aspartate receptor (NMDA-R) and α-amino-3-hydroxy-5-methyl-4-isoxazolepropionic acid receptor (AMPA-R).

Note that Table S1 in the supplementary material gives details of all models used in this work. However, we wish to highlight a simple transporter model based on Michaelis–Menten (MM) kinetics. The model uses extracellular Glu as the enzyme, similar to ATP transporter models. Using MM kinetics allows us to describe the EAAT with parameters that represent physiological conditions. Therefore, the EAAT is given:

3 $$\begin{array}{c}{I}_{eaat,psc}={V}_{eaat}ef{f}_{eaat}F\left(\frac{{\left[Glu\right]}_{ecs}}{{K}_{eaat}+{\left[Glu\right]}_{ecs}}\right)\end{array}$$

where *V*_*eaat*_ is the maximal EAAT velocity, *eff*_*eaat*_ is the average EAAT efficiency, *K*_*eaat*_ is the EAAT Glu affinity and [Glu]_*ecs*_ is the Glu concentration in the cleft.

In the model, Glu is released from the presynaptic ’active zone’ due to Ca^2+^ influx through VGCCs. Glu released during periods of neuronal activity activates postsynaptic Glu receptors and is cleared through diffusion and uptake by electrogenic EAATs on the surrounding PsC membrane. The rate of transmembrane Glu translocation by EAATs is relatively slow, approaching about 30 molecules of Glu per second^15,16^. The binding of Glu to the transporters (K_m_ ≈ 20 μM), is much faster, with a binding rate up to 10^7^ M^−1^ s^−1^^17^, and hence Glu transporters concentrated at the PsC provide for an almost instant buffering of Glu. The higher the density of transporters, the higher their buffering capacity^18^. It has been estimated that there are approximately 8000–10,000 EAATs per μm^2^ concentrated at the PsC^19^. The efficacy of EAAT Glu transport is about 50%^18^, meaning that approximately half of the Glu molecules dissociate from transporters and bind again to nearby receptors or other EAATs, which may affect the kinetics of Glu presence in the synaptic cleft. In this study, we fix the density of EAATs at 10,000 μm^−2^, the EAAT efficacy is 50% and, using Eq. (3) and based on the figures given, we calculate the time it takes the EAATs to clear 1 mM of Glu to be ∼33 ms. For each transport cycle, the EAAT co-transports 1Glu:3Na^+^:1H^+^ and counter-transports 1 K^+^, generating a PsC Na^+^ influx and K^+^ efflux (it should be noted that the proposed model does not account for the H^+^ fluxes or Cl^−^ which is thermodynamically uncoupled from Glu transport). The background activity of the NKA increases due to the intracellular Na^+^ and extracellular K^+^ concentrations changing from the respective transmembrane fluxes. Separate to K^+^ release through the EAAT, astrocytic K^+^ efflux is achieved through K_ir_4.1 channels densely populating perisynaptic membranes. The main synaptic K^+^ efflux pathway in the model is the current generated through the postsynaptic NMDA-R. Hodgkin-Huxley type voltage-gated K^+^ and Na^+^ channels are included for the generation of the presynaptic action potential. However, we assume the presynaptic voltage-gated K^+^ and Na^+^ channels do not interact with the synapse ‘active zone’^20^ therefore we exclude the contribution of these channels to the respective ionic concentration in the ECS.

**Figure 2:**
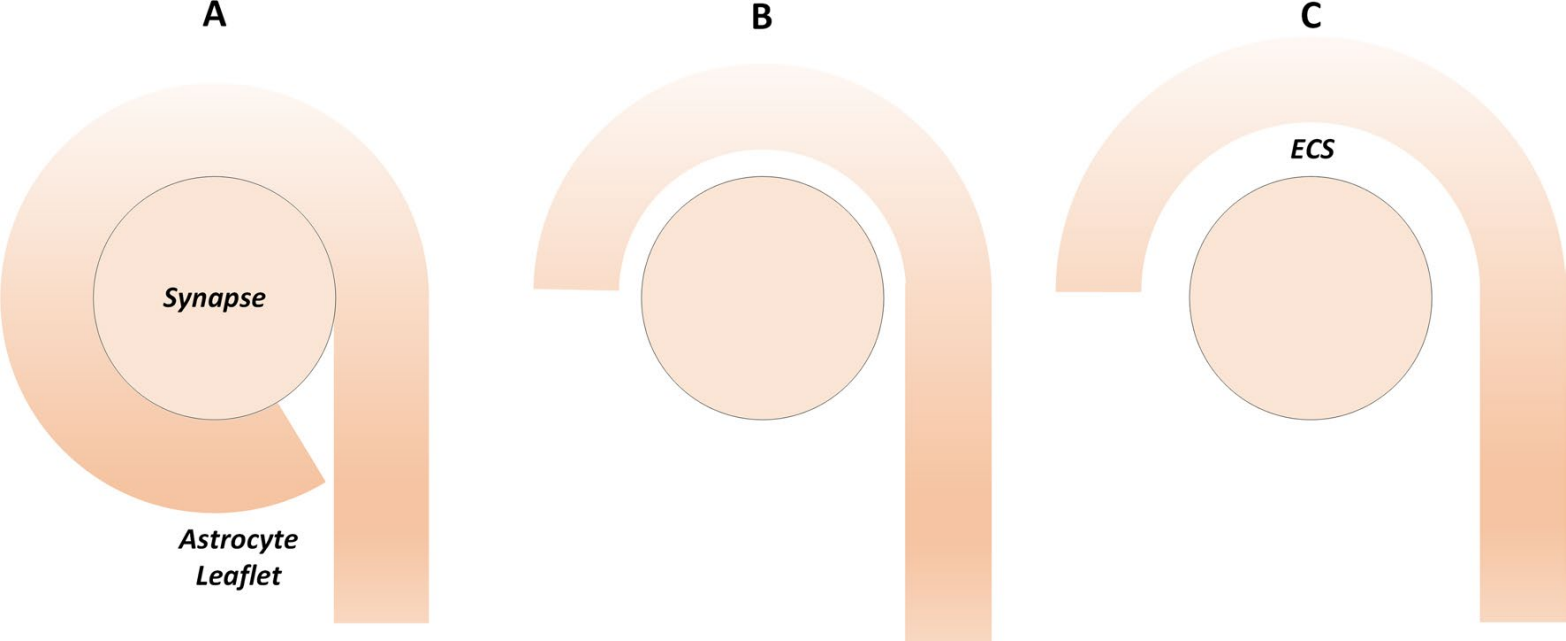
Schematic showing varying degrees of synaptic coverage by PsC. Three levels of PsC coverage represent the PsC in intimate contact with the synapse (**A**), when the PsC semi-wraps the synapse (**B**) and when the PsC is loosely coupled to the synapse (**C**). To simulate the various astrocyte-synapse couplings, the ECS volume and extracellular diffusion surface area is increased as the PsC moves away from the synapse.

To simulate the movement of the PsC during synaptic activation we carried out simulations for: (A) when the PsC is in intimate contact with the synapse and the ECS contains the cleft region only, (B) when a space exists between the PsC and the synapse such that the ECS volume is seven times that of the cleft and (C) when a space exists such that the ECS volume is approximately forty-nine times that of the cleft; Fig. 2 shows a schematic of the three levels of PsC coverage. The PsC and neuronal compartment volumes and surface areas are kept constant throughout all simulations.

The model was implemented using MATLAB 2019b (64-bit Windows version) by Mathworks. All simulation results presented in this section use a forward Euler numerical integration scheme with a fixed time step of t = 1 μs and all model simulations were carried out for 100 s. Note that ionic oscillations mediated through NCX activity^21–23^ are not visible in steady or dynamic states as the model does not track thermal fluctuations of individual ions. Instead, ionic concentrations are averaged at 1 μs time steps within each compartment of the model; in this work ionic diffusion within compartments is ignored as a well-mixed concentration is assumed. All transmembrane currents are given in femtoamps (fA), positive currents denote ions leaving the cellular compartment (negative ionic flux) and negative currents denote ions entering the cellular compartment (positive ionic flux).

## Results

We report the results on the time-dependent flow of ions between the PsC and the synaptic cleft with associated concentration dynamics. Prior to investigating how the spatial relationship between the synapse and the PsC affects ionic homeostasis, we establish our model for a fixed ECS volume. In the present case the ECS volume is fixed at 7 times the cleft volume (Fig. 2B), where the cleft volume is 0.0011 fL. In section "Varying levels of synaptic coverage by PsC" of this paper, we will consider ionic homeostasis for cases where the ECS is equal to the cleft volume (Fig. 2A) and where the ECS is forty-nine times the cleft volume (Fig. 2C).

Results are given as time series line plots that show the full 100 s simulation time. The last 100 ms of neuronal stimulation are given in the graph insets to show the finer detail of currents/concentrations.

### Neuronal activity

**Figure 3:**
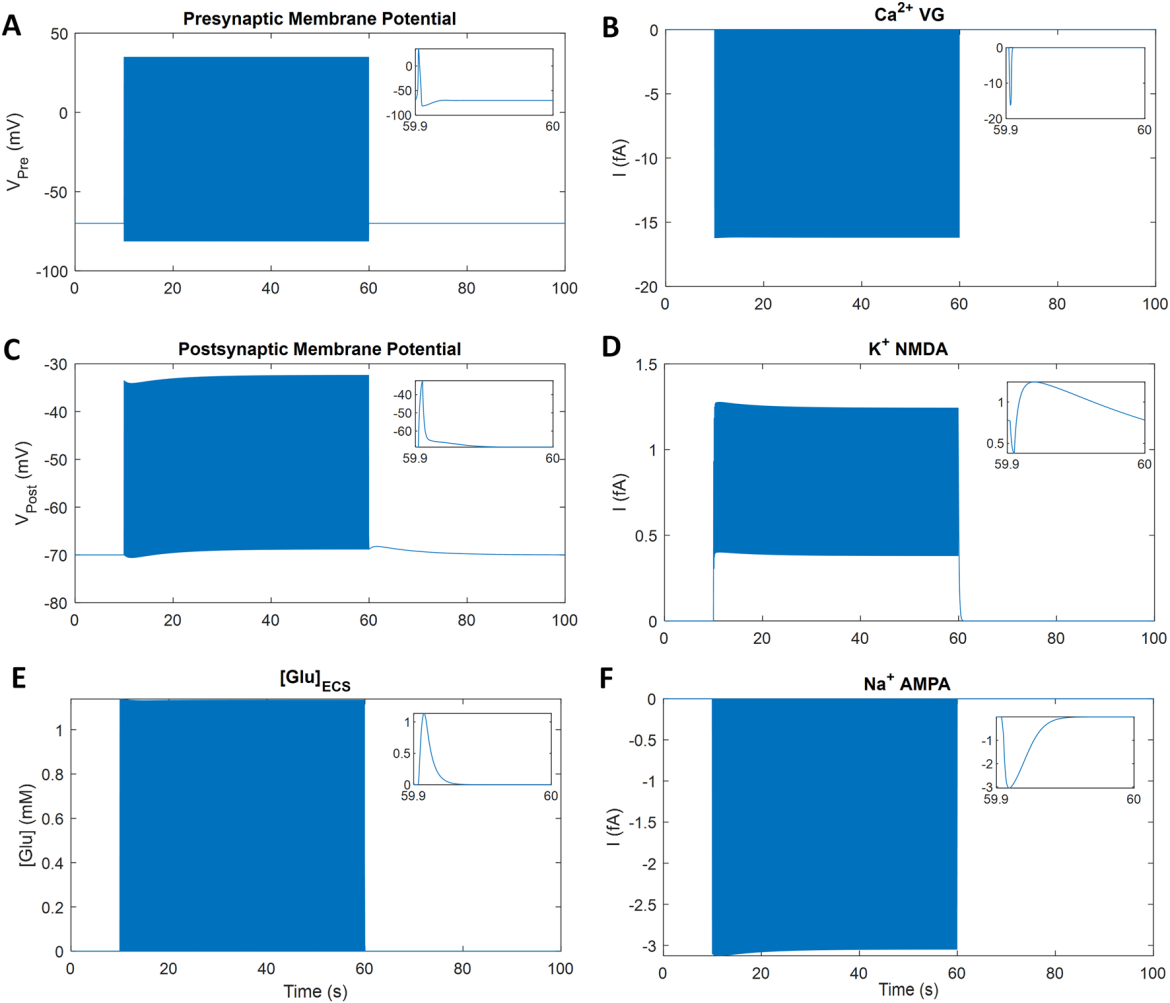
Simulation of neuronal activity. The inset shows the last 100 ms of the stimulus period. The action potential invades the presynaptic terminal, changing the membrane potential briefly (**A**), which allows the VGCCs to open (**B**) and subsequent Ca^2+^ influx promotes the release of Glu into the cleft (**E**). Glu activates the AMPA-R (**F**) and NMDA-R (**D**) on the Post, which causes a small depolarisation of the membrane potential (**C**).

The Pre is stimulated with an external current to produce a firing rate of 10 Hz for 50 s starting at t = 10 s, as shown in Fig. 3, with the graph insets showing the last 100 ms of stimulation. During each action potential, VGCCs open and the resulting Ca^2+^ influx (Fig. 3B) promotes Glu release into the synaptic cleft. Glu released from the Pre activates the Post receptors (AMPA-R and NMDA-R) and is cleared from the cleft after ∼33 ms (Fig. 3E inset). Activated AMPA-R channels typically allow Na^+^ into the Post (Fig. 3F) while K^+^ efflux into the synaptic cleft occurs through NMDA-R (Fig. 3D), subsequently K^+^ is taken up by the PsC by NKA. K^+^ released from the Pre is assumed to occur remote from the ECS space and is therefore ignored. The Pre action potential has a magnitude of approximately 100 mV; depolarising ∼90 mV and hyperpolarising ∼10 mV (Fig. 3A). To remove the NMDA-R magnesium block, the Post membrane is stimulated with a small external current to produce a 35 mV depolarisation (Fig. 3C) at the same time as the Pre Glu release. The AMPA-R current is larger than the NMDA-R current, however, the AMPA-R channel closes within 30 ms of activation by Glu while the NMDA-R closes within 300 ms of Glu activation. Therefore, the K^+^ release from the NMDA-R channel is slow compared to all other neuronal currents (Fig. 3D inset).

### Astrocytic homeostatic response

**Figure 4:**
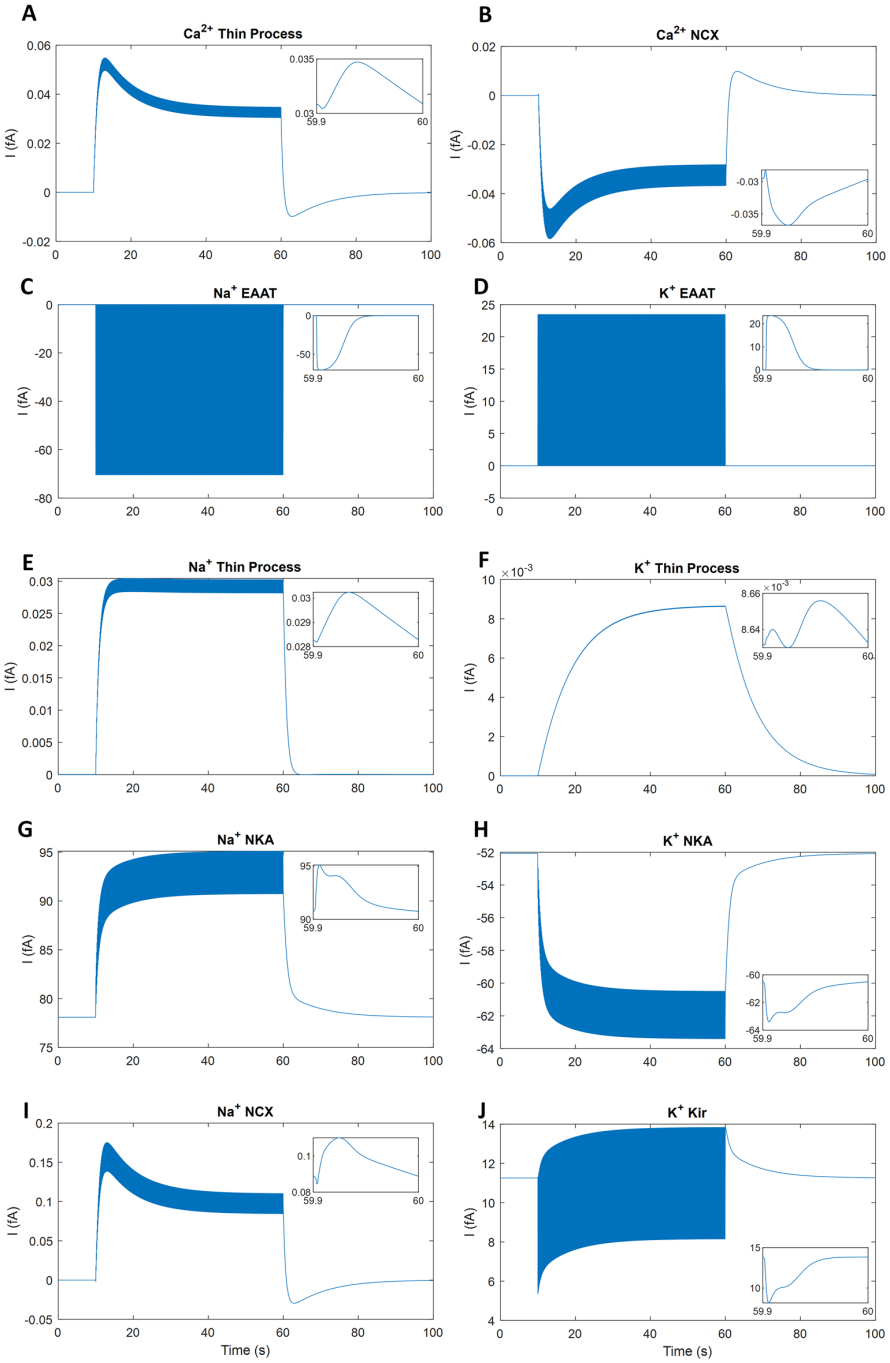
PsC homeostatic response currents. The inset shows the last 100 ms of the stimulus period and all currents are given in femtoamps (fA). The EAAT responds to ECS glutamate by transporting Na^+^ into the cradle (**C**) and transporting K^+^ out from the PsC (**D**). The perturbation of PsC and ECS ionic concentrations drives the NKA pump (**G**,**H**), NCX exchanger (**B**,**I**) and K_ir_4.1 channels (**J**) to maintain ionic homeostasis. The currents leaving the PsC through the thin process (**A**,**C**,**D**) are extremely small in comparison to all other currents. Note the passive background channel currents are not shown although they are included for model stability and completeness. We assume the PsC membrane is semi-permeable to K^+^, Na^+^ and Ca^2+^.

**Figure 5:**
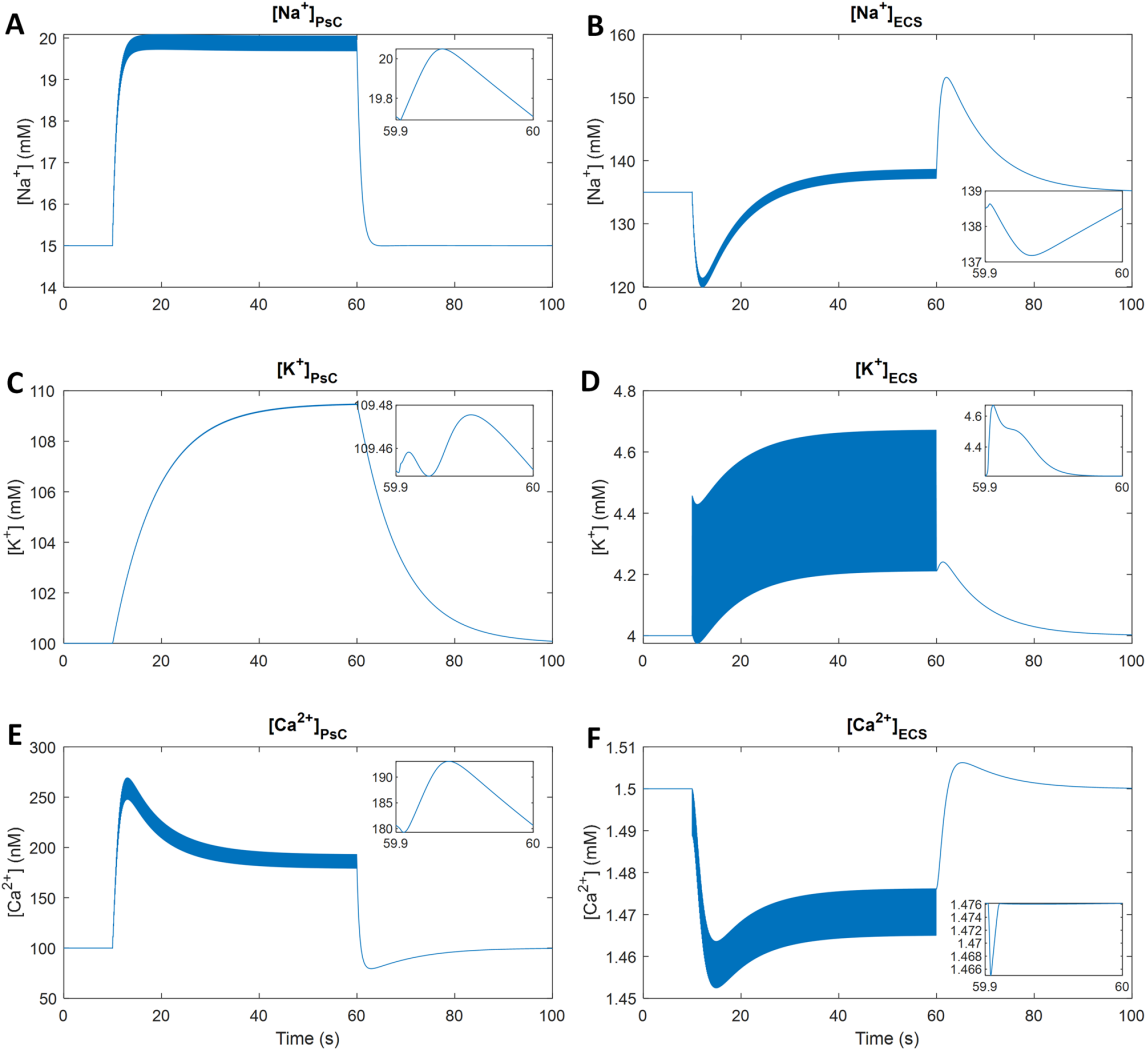
Astrocytic and extracellular ionic concentrations. The inset shows the last 100 ms of detail during the neuronal activity. Microdomains of Na^+^ (**A**), K^+^ (**C**) and Ca^2+^ (**E**) form in the PsC. Extracellular Na^+^ (**B**), K^+^ (**D**) and Ca^2+^ (**F**) concentrations and intracellular concentrations are interdependent.

Figure 4 shows the currents from the main astrocytic Ca^2+^, Na^+^ and K^+^ pathways through the EAAT, NKA, K_ir_4.1 and NCX. During neuronal stimulation, Glu released from the presynaptic cell binds and unbinds to the astrocytic EAATs. When the EAAT has completed its transport cycle and all Glu is cleared from the cleft, Na^+^ is taken up by the PsC and K^+^ is released into the ECS (Fig. 4C,D). Na^+^ taken up by the PsC causes the NKA pump activity to increase and the NCX reverses to extrude Na^+^ (Fig. 4G,I). At the same time, K^+^ released from the PsC through the K_ir_4.1 and EAAT (Fig. 4D,J) causes an increased current through the NKA pump (Fig. 4H). This ionic interplay leads to K^+^ and Na^+^ microdomain formation in the PsC (shown in Fig. 5A,B). The rise in both K^+^ and Na^+^ in the PsC is partly because of the extremely small flowing current down the thin leaflet; this is on the order of 8.5 × 10^–3^ fA for K^+^ and 0.03 fA for Na^+^ (Fig. 4E,F). On activation by Glu, the NMDA-R releases K^+^ over ∼300 ms and the EAAT transport lasts for ∼53 ms releasing K^+^ current from the PsC into the synaptic cleft; both increasing ECS K^+^ ([K^+^]_ECS_). The K_ir_4.1 is the main K^+^ exit pathway from the PsC and releases fewer K^+^ when the EAAT is active (Fig. 4J). The relatively large increase in [K^+^]_ECS_ causes the PsC NKA pump to increase K^+^ uptake. The PsC NCX acts on a timescale of tens of milliseconds, with the current rising ∼0.03 fA on every neural spike, and the NKA acts on a slightly longer timescale, hundreds of milliseconds, with the current rising ∼3 fA for each spike.

### Ionic concentrations

Figure 5 shows the ionic concentrations of K^+^, Na^+^ and Ca^2+^ in the PsC and ECS compartments. The NKA on the PsC membrane prevents the ECS from being flooded with K^+^ from the NMDA-R, EAAT and K_ir_4.1. K^+^ taken up by the astrocytic NKA is dominant over the K^+^ release from EAAT, K_ir_4.1 and leakage through the membrane, therefore, a small microdomain starts forming in the PsC; ∼9.5 mM over 50 s of neuronal activity (Fig. 5C). After the sustained neuronal activity has ceased, K^+^ stored in the PsC is released back into the ECS slowly mainly through the K_ir_4.1 and leak channels. The interplay between K^+^ uptake by the NKA on the Pre and K^+^ efflux from the PsC causes [K^+^]_ECS_ in the ECS to become slightly elevated for ∼20 s after the neuronal activity has ceased (Fig. 5D). K^+^ also leaves the ECS through electrochemical diffusion into the GECS (data not shown). The K^+^ microdomain dissipates slowly because the astroglial K_ir_4.1 channel releases K^+^ slowly (Fig. 4H). This result demonstrates that K^+^ released into the ECS via NMDA-R is taken up by the PsC via NKA activity and then released back into the ECS primarily by K_ir_4.1 to be taken up again by the presynaptic neuron via NKA. Relatively large Na^+^ and Ca^2+^ microdomains form in the PsC due to Na^+^ influx through EAAT and Ca^2+^ influx through the reversal of the NCX. The Na^+^ and Ca^2+^ microdomains dissipate within seconds after neuronal activity ceases (Fig. 5A,E); this action is driven primarily by the forward mode of the NCX for Ca^2+^ efflux and by the NKA for Na^+^ efflux. The mode of NCX operation is sensitive to the intracellular and extracellular Na^+^ concentration, thus the PsC Ca^2+^ microdomain is driven primarily by the combination of PsC and ECS Na^+^ concentrations. The Ca^2+^ microdomain reaches ∼150 nM above baseline levels. Once the neuronal activity has ceased and the Na^+^ microdomain dissipates from the PsC, the NCX extrudes Ca^2+^ in the forward mode which causes the ECS Ca^2+^ ([Ca^2+^]_ECS_) to overshoot slightly and then return to baseline concentration (Fig. 5F). The EAAT removes ∼2.5 mM of Na^+^ from the ECS for every glutamate release event, which corresponds to ∼0.6 mM increase in Na^+^ in the PsC ([Na^+^]_PsC_) and this leads to the PsC Na^+^ microdomain (Fig. 5A). During the sustained period of activity simulated, Na^+^ in the ECS ([Na^+^]_ECS_) reached a steady-state after ∼30 s; with a minimum of ∼15 mM below the baseline before returning to baseline and finally overshooting by ∼20 mM when neural activity ceases (Fig. 5B). This is due to the increased activity of both the NKA and NCX where their associated Na^+^ effluxes become comparable to the EAAT Na^+^ influx (Fig. 4). The PsC Na^+^ microdomain subsequently reaches steady-state ∼5 mM above baseline due to the activity of the EAAT being counteracted by both the NKA and NCX. The activity of the NKA is dependent on a combination of the PsC Na^+^ and ECS K^+^ concentrations (assuming there is sufficient ATP). Through interactions with PsC and ECS Na^+^, K^+^ and Ca^2+^ are, indirectly, functionally coupled.

### Varying levels of synaptic coverage by PsC

**Figure 6:**
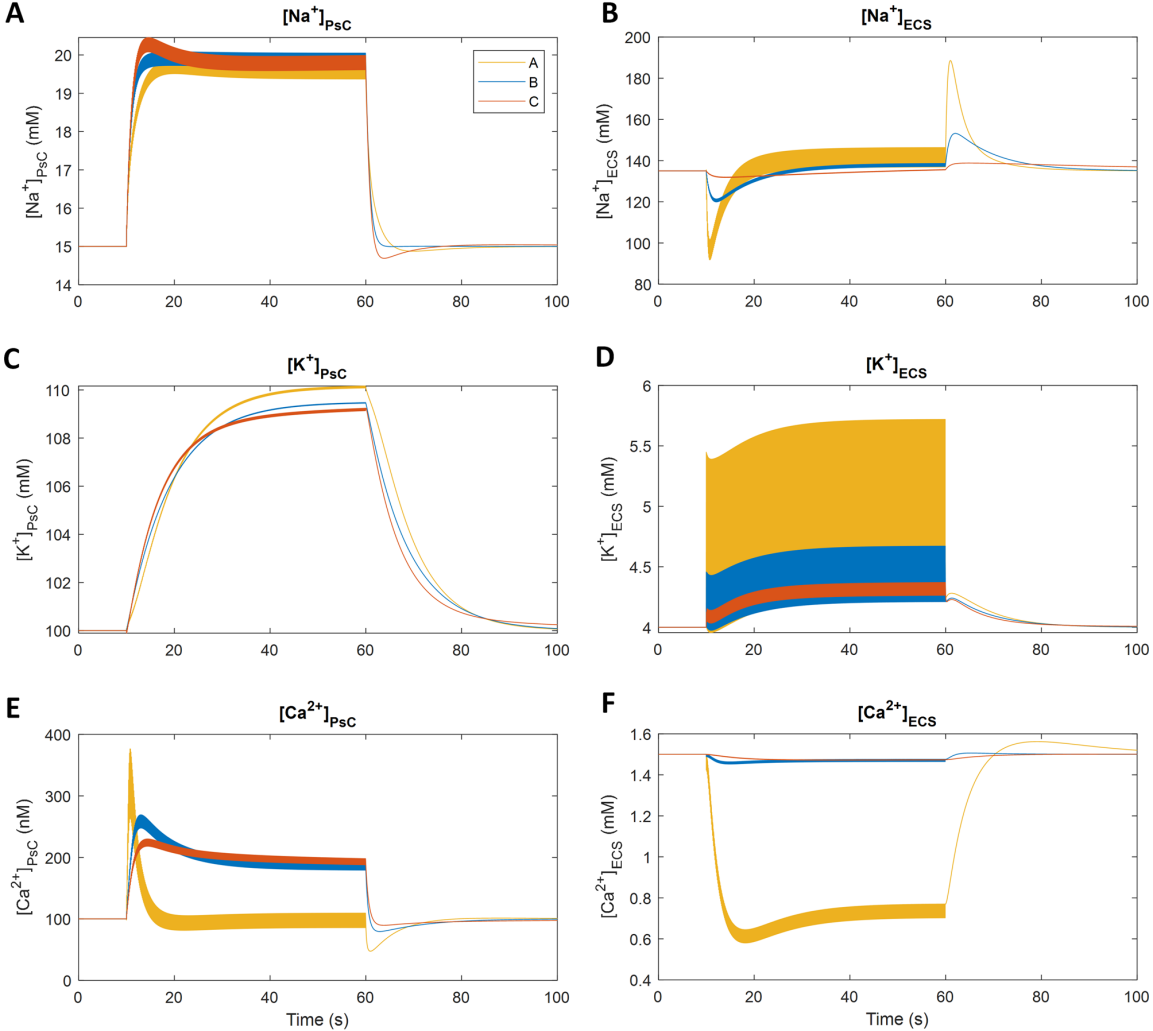
Comparison of ionic concentrations with variable degrees of PsC coverage of the neuronal synapse. The concentrations of Na^+^, K^+^ and Ca^2+^ in the PsC are shown in the left-hand column (**A**,**C**,**E**) and the ionic concentrations in the ECS are shown in the right-hand column (**B**,**D**,**F**). (**A**) (yellow line) is when the ECS volume is equal to the cleft volume, representing a synapse in direct contact with a PsC. (**B**) (blue line) is the ECS volume used in all previous model simulations, which is seven times the cleft volume, representing a semi-enwrapped synapse. (**C**) (orange line) is when the ECS volume is forty-nine times that of the cleft, representing a synapse with a relatively distant PsC.

Figure 6 shows a comparison of the ionic concentrations between the various levels of PsC coverage and the ECS volume-dependent results used in the previous simulations are shown in blue for ease of comparison. Ionic concentrations are changing depending on the ECS volume. As expected, the exchange of ions between the PsC and the ECS is much more pronounced when these two regions are in intimate contact (Fig. 2A). As the distance between the PsC and synapse increases (Fig. 2B,C), this ionic exchange appears to be less dependent on the ECS volume. The K^+^ and Na^+^ concentrations in the ECS are significantly increased for coupling A, while the Ca^2+^ in the cleft drops to approximately half the baseline concentration before rapid recovery due to the PsC membrane-bound NCX switching to forward mode: note that we attribute the rapid drop in Ca^2+^ in the ECS (Fig. 6F) to the inability of diffusion from the GECS to replenish Ca^2+^ removed from the ECS by the reversal of PsC membrane-bound NCX and Pre/Post uptake. As the distance increases, diffusion of ions from the GECS to the ECS increases and the concentrations of ions in the ECS can be maintained closer to baseline throughout the stimulus. The ionic concentrations in the PsC for K^+^ and Ca^2+^ are noticeably impacted when the PsC is in intimate contact with the synapse. However, Na^+^ appears to be relatively independent of the PsC-synapse distance relationship because the amount of glutamate released at each spike is constant. Therefore, the density of EAAT transporters remains the same and consequently the amount of Na^+^ transported into the PsC will be relatively independent of the ECS volume.

## Discussion

The synaptic cradle model was used to simulate how astrocyte leaflet motility would affect ionic shuttling dynamics at an active synapse. The results from model simulations show that astrocytes clear extracellular K^+^ through NKA, but because of the slow restricted pathway through the ultra-thin leaflet, most of the K^+^ ions reside long enough in the PsC to be returned to the cleft by K_ir_4.1. This restricted pathway through the thin leaflet is unlikely to be pure electrodiffusion but rather due to ionic retention sites as a result of tortuosity, and also negative fixed membrane charge may offer some restriction to ionic flow due to attraction. Therefore, K^+^ ions are transiently stored in the PsC and then returned to the cleft. The simulations also show that Na^+^ and Ca^2+^ microdomains form in the cradle where the former is due to the dominance of EAAT Na^+^ influx over NKA, NCX mediated Na^+^ efflux. Note that the Na^+^ current flowing away from the PsC towards the branch is relatively insignificant due to the retention of positive ions along the leaflet^24^. A direct result of the elevated [Na^+^]_PsC_ in the cradle is the influx of Ca^2+^ through the reversed NCX giving rise to the Ca^2+^ microdomain. Both the Na^+^ and Ca^2+^ microdomains rapidly dissipate after neuronal excitation because NKA activity produces a net efflux of Na^+^ from the PsC. As the [Na^+^]_PsC_ drops, the NCX returned to a forward mode and Ca^2+^ is exported to the ECS. However, the K^+^ microdomain in the PsC is much slower to dissipate because K^+^ ions are continually being shuttled between the ECS and PsC: K^+^ is continually taken up by the PsC membrane-bound NKA and leaks back to the ECS by K_ir_4.1 and K^+^ leak channels.

Varying degrees of PsC coverage of the synapse show that the smaller the ECS volume, the stronger the influence of the PsC on ionic signalling and homeostasis at the synapse. The [K^+^]_ECS_ and [Na^+^]_ECS_ changes are more pronounced and the [Ca^2+^]_ECS_ drops to approximately half the resting concentration when there is no diffusion of Ca^2+^ from the GECS. The PsC K^+^ ([K^+^]_PsC_) and [Na^+^]_PsC_ show a similar trend regardless of the volume. However, the PsC K^+^ microdomain is slightly larger when the PsC is in intimate contact with the synapse as less K^+^ can efflux from the ECS to the GECS and there is a build-up of [K^+^]_ECS_. The PsC Ca^2+^ ([Ca^2+^]_PsC_) increases initially but after approximately 10 s of synaptic activity, it returns to the resting concentration and thereafter toggles around that level until the neuronal activity has ceased. This drop in [Ca^2+^]_PsC_ is a consequence of the significant drop in [Ca^2+^]_ECS_ which forces the NCX into a forward mode. After the neuronal activity has ceased the [Ca^2+^]_PsC_ drops below the resting concentration due to the NCX entering forward mode and extruding Ca^2+^ from the PsC, however, [Ca^2+^]_PsC_ immediately returns to baseline due to leakage through the membrane.

A noteworthy prediction from the model is that a PsC that tightly enwraps the synapse lacks the ability to retain a Ca^2+^ microdomain whereas when the PsC is remote from the synapse a Ca^2+^ microdomain forms in the PsC. This is supported by evidence highlighting that astrocytes can regulate Ca^2+^ transients in spatially distinct compartments^25^. This spatial separation suggests that Ca^2+^ microdomains may be involved in PsC-directed motility, and evidence supports a role for [Ca^2+^]_PsC_ in mediating peripheral astrocyte process extension (and possibly retraction) through interactions between profilin-1 and actin^26^. Furthermore Ca^2+^ transients have been shown to mediate perisynaptic astrocytic process motility in the hippocampus^27^. Therefore, the formation of a Ca^2+^ microdomain may cause the PsC to move towards the synapse initially but this movement is regulated as the Ca^2+^ microdomain diminishes when the Ca^2+^ extracellular diffusion becomes insignificant. These observations have implications on astrocytic ionic contributions to synaptic stabilisation, where astrocytic Ca^2+^ has been proposed to play a pivotal role^27^. This level of organisation requires the generation of ionic microdomains, which have been reported experimentally^3,28^. Data presented here supports this, but also adds an important consideration of the dynamics of these microdomains as they approach and interact with synapses.

In summary, the computational modelling of astrocytic ionic homeostasis has revealed novel, localised storage solutions, in the form of microdomains, with respect to K^+^ buffering. In addition, the predictions allude to a differential role for perisynaptic astrocytic [Ca^2+^]_PsC_ in engaging with and stabilising synapses. There is evidence to support these data in existing biological datasets, but the true in vivo extent of their interplay remains to be resolved. These data therefore further our understanding and enhance the role that astrocytic perisynaptic processes play in regulating synaptic physiology and architecture.

## Supplementary Information

Supplementary Information.

## Data Availability

The model code used in this study is available in the author’s GitHub repository at: https://github.com/MarinusToman/astroglial-shuttling/.
