# Supplementary material for: The influence of astrocytic leaflet motility on ionic signalling and homeostasis at active synapses
Source: Sci Rep. 2023 Feb 21;13:3050. doi: 10.1038/s41598-023-30189-8 (PMC9944253; doi:10.1038/s41598-023-30189-8)
Supplement: Supplementary file 1 — Supplementary Information. [file 41598_2023_30189_MOESM1_ESM.pdf]

The influence of astrocytic leaflet motility on ionic signalling and homeostasis at active synapses – Supplementary Material

Marinus Toman [1*], John Joseph Wade [1], Alexei Verkhratsky [2,3,4], Mark Dallas [5], Angela Bithell [5], Bronac Flanagan [1], Jim Harkin [1] and Liam McDaid [1]

[1] Computational Neuroscience and Neuromorphic Engineering Team, Intelligent Systems Research Centre, Ulster University, Northland Road, Derry, United Kingdom BT48 7JL

[2] Faculty of Biology, Medicine and Health, University of Manchester, Manchester, United Kingdom

[3] Achucarro Center for Neuroscience, IKERBASQUE, Basque Foundation for Science, Bilbao, Spain

[4] Department of Stem Cell Biology, State Research Institute Centre for Innovative Medicine, LT-01102, Vilnius, Lithuania

[5] Reading School of Pharmacy, University of Reading, Reading, United Kingdom

[*] Corresponding author

Table 1. Transmembrane currents used in the synaptic cradle model.

Current	Description	Equation(s)	Source
$\boldsymbol{I_{vgnc,pre}}$	Voltage-gated Na^+ channel	$g_{vgnc,pre}m^3h(V_{pre} - E_{Na})$	1
$\boldsymbol{I_{vgkc,pre}}$	Voltage-gated K^+ channel	$g_{vgkc,pre}n^4(V_{pre} - E_K)$	1
$\boldsymbol{I_{l,pre}}$	Background leak channel	$g_{l,pre}(V_{pre} - E_l)$	1
$\boldsymbol{I_{vgcc,pre}}$	Voltage-gated Ca^{2+} channel	$g_{vgcc,pre}s(V_{pre} - E_{Ca})$	2
$\boldsymbol{\frac{dw}{dt}}$	Voltage-gated (in)activation	$\alpha_w(1-w) - \beta_w w$	1
$\boldsymbol{\alpha_m}$	Na^+ activation forward rate	$0.1\left(\frac{25 - V_{pre}}{exp\left(\frac{25 - V_{pre}}{10}\right) - 1}\right)$	Adapted from [1]
$\boldsymbol{\beta_m}$	Na^+ activation backward rate	$4\left(\frac{-V_{pre}}{18}\right)$	Adapted from [1]
$\boldsymbol{\alpha_h}$	Na^+ inactivation forward rate	$0.07\ exp\left(\frac{-V_{pre}}{20}\right)$	Adapted from [1]
$\boldsymbol{\beta_h}$	Na^+ inactivation backward rate	$\left(exp\left(\frac{30 - V_{pre}}{10}\right) + 1\right)^{-1}$	Adapted from [1]
$\boldsymbol{\alpha_n}$	K^+ activation forward rate	$0.1\left(\frac{10 - V_{pre}}{exp\left(\frac{10 - V_{pre}}{10}\right) - 1}\right)$	Adapted from [1]
$\boldsymbol{\beta_n}$	K^+ activation backward rate	$0.125\left(\frac{-V_{pre}}{80}\right)$	Adapted from [1]
$\boldsymbol{\alpha_s}$	Ca^{2+} activation forward rate	$\frac{1.6}{1 + exp\left(-0.072(V_{pre} - 5)\right)}$	Adapted from [2]
$\boldsymbol{\beta_s}$	Ca^{2+} activation backward rate	$\frac{0.02(V_{pre} - 1.31)}{exp\left(\frac{V_{pre} - 1.31}{5.36}\right) - 1}$	Adapted from [2]
$\boldsymbol{I_{pmca,pre}}$	Plasma-membrane Ca^{2+}-ATPase (PMCA)	$\bar{I}_{pmca}\left(\frac{[Ca^{2+}]_{pre}}{K_{Ca,pre} + [Ca^{2+}]_{pre}}\right)$	3
$\boldsymbol{I_{nka,y}}$	Na^+-K^+-ATPase (NKA)	$\bar{I}_{nka}\left(\frac{[Na^+]_y^{1.5}}{K_{Na,y}^{1.5} + [Na^+]_y^{1.5}} \times \frac{[K^+]_y}{K_{K,y} + [K^+]_y}\right)$	4
$\boldsymbol{I_{ampa,post}}$	AMPA receptor	$g_{ampa}r(V_{post} - E_{ampa})$	5
$\boldsymbol{I_{nmda,post}}$	NMDA receptor	$g_{ampa}B(V_{post})r(V_{post} - E_{ampa})$	5

Current	Description	Equation(s)	Source
$\frac{dr}{dt}$	AMPA/NMDA (in)activation	$\alpha_r [Glu]_{ecs}(1-r) - \beta_r r$	5
$B(V_{post})$	NMDA magnesium block	$\left(1 + exp(-0.062V_{post})\frac{[Mg]_o}{3.57}\right)^{-1}$	6
$I_{eaat,psc}$	Excitatory amino-acid transporter (EAAT)	$\bar{I}_{eaat}\left(\frac{[Glu]_{ecs}}{K_{eaat}+[Glu]_{ecs}}\right)$	-
$I_{ncx,psc}$	Na^+/Ca^{2+} exchanger (NCX)	$\bar{I}_{ncx}\left(\left(\frac{[Na^+]_{psc}}{[Na^+]_{ecs}}\right)^3 exp\left(\frac{\gamma F V_{psc}}{RT}\right) - \left(\frac{[Ca^{2+}]_{psc}}{[Ca^{2+}]_{ecs}}\right) exp\left(\frac{(\gamma-1)FV_{psc}}{RT}\right)\right)$	4,7
$I_{kir,psc}$	K^+ inward rectifier channel (K_{ir}4.1)	$g_{kir}\sqrt{[K^+]_{ecs}}(V_{psc}-E_{kir})$	8
$I_{pf,x}$	Leaflet diffusion	$K_{pf,x}\frac{-E_x}{l_{lf}}\ exp\left(-\frac{Q\left(\varphi - \sqrt{\frac{Q(-E_x)}{l_{lf}\pi\epsilon}}\right)}{k_B T}\right)$	Adapted from 9
$I_{l,x,y}$	Passive channels	$g_{l,x,y}(V_y - E_x)$	3
$I_{ecs,x}$	ECS diffusion	$g_{ecs}\lambda(-E_{x,ecs})$	10
E_x	Equilibrium potential	$\frac{RT}{z_x F} ln\frac{[x]_o}{[x]_i}$	3
**Note:** $w \in \{m,h,n,s\}, x \in \{Na,K,Ca\}$ and $y \in \{pre,post,psc\}$			

Table 2. Parameters used in the synaptic cradle model.

Parameter	Description	Value	Unit(s)	Source
**Constants**				
dt	Time step	10	µs	-
F	Faraday’s constant	96485	C/mol	-
R	Idela gas constant	8.314	J/mol.K	-
k_B	Boltzmann’s constant	1.38×10^{-23}	J/K	-
T	Absolute temperature	310	K	-
Q	Elementary charge	1.6002×10^{-19}	C	-
z_K	Valency of K^+	1	-	-
z_{Na}	Valency of Na^+	1	-	-
z_{Ca}	Valency of Ca^{2+}	2	-	-
**Neuronal parameters**				

Parameter	Description	Value	Unit(s)	Source
V_{pre}	Presynaptic membrane potential	-70	mV	-
V_{post}	Postsynaptic membrane potential	-70	mV	-
C_m	Membrane capacitance	0.01	F/m^2	1
$g_{vgnc,pre}$	Maximal Na^+ conductance	120	mS/cm^2	1
E_{Na}	Na^+ equilibrium potential	45	mV	Adapted from [1]
$g_{vgkc,pre}$	Maximal K^+ conductance	36	mS/cm^2	1
E_K	K^+ equilibrium potential	-82	mV	Adapted from [1]
$g_{vgcc,pre}$	Maximal Ca^{2+} conductance	0.1	mS/cm^2	Adapted from [2]
E_{Ca}	Ca^{2+} equilibrium potential	$\frac{RT}{z_{Ca}F} ln \frac{[Ca^{2+}]_{ecs}}{[Ca^{2+}]_{pre}}$	V	-
$g_{l,pre}$	Maximal leak conductance	0.3	mS/cm^2	1
E_l	Leak equilibrium potential	-59	mV	Adapted from [1]
V_{max}	Maximal PMCA velocity	0.2×10^{-6}	mol/m^2s	3
$\bar{I}_{pmca}$	Maximal PMCA current	$V_{max}F$	A/m^2	3
$K_{Ca,pre}$	PMCA Ca^{2+} affinity	0.2	µM	3
P_{max}	Maximal NKA velocity	1.12×10^{-2}	mol/m^2s	4
$\bar{I}_{nka}$	Maximal NKA current	$P_{max}F$	A/m^2	4
$K_{Na,pre/post}$	NKA Na^+ affinity	10	mM	14
$K_{K,pre/post}$	NKA K^+ affinity	0.6	mM	14
g_{ampa}	Maximal AMPA conductance	0.18	S/m^2	5
E_{ampa}	AMPA equilibrium potential	0	mV	5
α_{ampa}	AMPA activation rate	1.1×10^6	$M^{-1}s^{-1}$	5
β_{ampa}	AMPA inactivation rate	190	s^{-1}	5
g_{nmda}	Maximal NMDA conductance	0.26	S/m^2	6
E_{nmda}	NMDA equilibrium potential	0	mV	6
α_{nmda}	NMDA activation rate	7.4×10^4	$M^{-1}s^{-1}$	6
β_{nmda}	NMDA inactivation rate	6.6	s^{-1}	6
$g_{l,Na,pre}$	Na^+ leak conductance	1.4184	S/m^2	Calculated
$g_{l,K,pre}$	K^+ leak conductance	7.6113	S/m^2	Calculated
$g_{l,Ca,pre}$	Ca^{2+} leak conductance	0.0186	S/m^2	Calculated
$g_{l,Na,post}$	Na^+ leak conductance	1.4184	S/m^2	Calculated
$g_{l,K,post}$	K^+ leak conductance	7.6113	S/m^2	Calculated
$g_{l,Ca,post}$	Ca^{2+} leak conductance	0.0186	S/m^2	Calculated
**Astrocytic parameters**				

Parameter	Description	Value	Unit(s)	Source
$\boldsymbol{V_{psc}}$	Membrane potential	-80.7	mV	10
$\boldsymbol{V_{eaat}}$	Maximal EAAT velocity	3 x 10^{-6}	mol/m^2s	11
$\boldsymbol{eff_{eaat}}$	Average EAAT efficiency	0.5	-	12
$\boldsymbol{\bar{I}_{eaat}}$	Maximal EAAT current	$V_{eaat} eff_{eaat} F$	A/m^2	-
$\boldsymbol{K_{eaat}}$	EAAT Glu affinity	20	µM	13
$\boldsymbol{K_{Na,psc}}$	NKA Na^+ affinity	10	mM	14
$\boldsymbol{K_{K,psc}}$	NKA K^+ affinity	3.6	mM	14
$\boldsymbol{\bar{I}_{ncx}}$	Maximal NCX current	1	A/m^2	7
$\boldsymbol{\gamma}$	NCX energy partition	0.5	-	7
$\boldsymbol{g_{kir}}$	Maximal K_{ir}4.1 conductance	144	S/m^2	10
$\boldsymbol{E_{kir}}$	K_{ir}4.1 equilibrium potential	$\frac{RT}{z_K F} ln \frac{[K^+]_{ecs}}{[K^+]_{psc}}$	V	-
$\boldsymbol{K_{pf}}$	Poole-Frenkel channel constant	0.018	S/m	9
$\boldsymbol{\varphi}$	Well activation energy	10	J	9
$\boldsymbol{\epsilon}$	Dynamic permittivity	$\epsilon_0 \epsilon_r$	-	9
$\boldsymbol{\epsilon_0}$	Vacuum permittivity	8.85 x 10^{-12}	F/m	9
$\boldsymbol{\epsilon_r}$	Relative permittivity of brain tissue	0.82	F/m	9
$\boldsymbol{g_{l,Na,psc}}$	Na^+ leak conductance	2.3771	S/m^2	Calculated
$\boldsymbol{g_{l,K,psc}}$	K^+ leak conductance	33.0159	S/m^2	Calculated
$\boldsymbol{g_{l,Ca,psc}}$	Ca^{2+} leak conductance	1.6 x 10^{-11}	S/m^2	Calculated
**Extracellular parameters**				
$\boldsymbol{g_{ecs}}$	Maximal diffusion conductance	1	S/m^2	10
$\boldsymbol{\lambda}$	Conductance scaling factor	10	-	-
$\boldsymbol{E_{x,ecs}}$	ECS equilibrium potential	$\frac{RT}{z_x F} ln \frac{[x]_{gecs}}{[x]_{ecs}}$	V	10
**Concentrations**				
$\boldsymbol{[K^+]_{psc}}$	Initial K^+ in PsC	100	mM	14
$\boldsymbol{[K^+]_{ecs}}$	Initial K^+ in ECS	4	mM	14
$\boldsymbol{[K^+]_{pre}}$	K^+ in Pre	100	mM	14
$\boldsymbol{[K^+]_{post}}$	K^+ in Post	100	mM	14
$\boldsymbol{[K^+]_{gecs}}$	K^+ in GECS	4	mM	14
$\boldsymbol{[Na^+]_{psc}}$	Initial Na^+ in PsC	15	mM	14
$\boldsymbol{[Na^+]_{ecs}}$	Initial Na^+ in ECS	135	mM	14
$\boldsymbol{[Na^+]_{pre}}$	Na^+ in Pre	15	mM	14

Parameter	Description	Value	Unit(s)	Source
$[Na^{+}]_{post}$	Na^{+} in Post	15	mM	14
$[Na^{+}]_{gecs}$	Na^{+} in GECS	135	mM	14
$[Ca^{2+}]_{psc}$	Initial Ca^{2+} in PsC	100	nM	14
$[Ca^{2+}]_{ecs}$	Initial Ca^{2+} in ECS	1.5	mM	14
$[Ca^{2+}]_{pre}$	Initial Ca^{2+} in Pre	50	nM	14
$[Ca^{2+}]_{post}$	Ca^{2+} in Post	50	nM	14
$[Ca^{2+}]_{gecs}$	Ca^{2+} in GECS	1.5	mM	14
**Morphology parameters**				
Vol_{psc}	PsC volume	0.031416	fL	Calculated
Vol_{pre}	Pre volume	0.014314	fL	Calculated
Vol_{post}	Post volume	0.014314	fL	Calculated
$Vol_{ecs,a}$	ECS volume (A configuration)	0.001145	fL	Calculated
$Vol_{ecs,b}$	ECS volume (B configuration)	0.00786	fL	Calculated
$Vol_{ecs,c}$	ECS volume (C configuration)	0.055022	fL	Calculated
SA_{psc}	PsC surface area	0.23562	μm^2	Calculated
SA_{pre}	Pre surface area	0.21206	μm^2	Calculated
SA_{post}	Post surface area	0.21206	μm^2	Calculated
SA_{ecs}	ECS diffusion surface area	0.015	μm^2	Calculated
CSA_{lf}	Leaflet cross-sectional area	0.007854	μm^2	Calculated
l_{lf}	Leaflet length	2	μm	-

References

1. Hodgkin, A. L. & Huxley, A. F. A quantitative description of membrane current and its application to conduction and excitation in nerve. *J. Physiol.* **117**, 500–44 (1952).

2. Mirzakhalili, E., Epureanu, B. I. & Gourgou, E. A mathematical and computational model of the calcium dynamics in Caenorhabditis elegans ASH sensory neuron. *PLoS One* **13**, e0201302 (2018).

3. Keener, J. & Sneyd, J. *Mathematical Physiology*. vol. 8/1 (Springer New York, 2009).

4. Gabbiani, F. & Cox, S. J. *Mathematics for Neuroscientists*. *Mathematics for Neuroscientists* (Elsevier, 2010). doi:10.1016/C2009-0-01867-4.

5. Destexhe, A. Kinetic Models of Postsynaptic Currents. in *Encyclopedia of Computational Neuroscience* 1–6 (Springer New York, 2014). doi:10.1007/978-1-4614-7320-6_355-1.

6. Jahr, C. & Stevens, C. Voltage dependence of NMDA-activated macroscopic conductances predicted by single-channel kinetics. *J. Neurosci.* **10**, 3178–3182 (1990).

7. Sterratt, D., Graham, B., Gillies, A. & Willshaw, D. *Principles of Computational Modelling in Neuroscience*. (2011).

8. Witthoft, A., Filosa, J. A. & Karniadakis, G. E. Potassium buffering in the neurovascular unit: models and sensitivity analysis. *Biophys. J.* **105**, 2046–54 (2013).

9. Breslin, K. *et al.* Potassium and sodium microdomains in thin astroglial processes: A computational model study. *PLOS Comput. Biol.* **14**, e1006151 (2018).

10. Wade, J. J. *et al.* Calcium Microdomain Formation at the Perisynaptic Cradle Due to NCX Reversal: A Computational Study. *Front. Cell. Neurosci.* **13**, (2019).

11. Marcaggi, P. & Attwell, D. Role of glial amino acid transporters in synaptic transmission and brain energetics. *Glia* **47**, 217–225 (2004).

12. Vandenberg, R. J. & Ryan, R. M. Mechanisms of Glutamate Transport. *Physiol. Rev.* **93**, 1621–1657 (2013).

13. Tzingounis, A. V. & Wadiche, J. I. Glutamate transporters: confining runaway excitation by shaping synaptic transmission. *Nat. Rev. Neurosci.* **8**, 935–947 (2007).

14. Verkhratsky, A. & Nedergaard, M. Physiology of Astroglia. *Physiol. Rev.* **98**, 239–389 (2018).
